# Laboratory cohabitation challenge model for shrimp hepatopancreatic microsporidiosis (HPM) caused by *Enterocytozoon hepatopenaei* (EHP)

**DOI:** 10.1186/s12917-016-0923-1

**Published:** 2017-01-05

**Authors:** Paul Vinu Salachan, Pattana Jaroenlak, Siripong Thitamadee, Ornchuma Itsathitphaisarn, Kallaya Sritunyalucksana

**Affiliations:** 1 Shrimp–pathogen interaction (SPI) laboratory, National Center for Genetic Engineering and Biotechnology (BIOTEC), National Science and Technology Development Agency (NSTDA), Yothi office, Bangkok, 10400 Thailand; 2 Center of Excellence for Shrimp Molecular Biology and Biotechnology, Faculty of Science, Mahidol University, Rama VI Rd., Bangkok, 10400 Thailand; 3 Department of Biochemistry, Faculty of Science, Mahidol University, Rama VI Rd., Bangkok, 10400 Thailand; 4 Department of Biotechnology, Faculty of Science, Mahidol University, Rama VI Rd., Bangkok, 10400 Thailand

**Keywords:** *Enterocytozoon hepatopenaei*, Shrimp microsporidian, Cohabitation assay, *Penaeus vannamei*

## Abstract

**Background:**

*Enterocytozoon hepatopenaei* (EHP) causes hepatopancreatic microsporidiosis (HPM) in shrimp. It is probably endemic in Australasia and was first characterized and named from the giant or black tiger shrimp *Penaeus monodon* from Thailand in 2009. Later, it was also found to infect exotic *Penaeus vannamei* imported for cultivation in Asia. HPM is not normally associated with shrimp mortality, but information from shrimp farmers indicates that it is associated with significant growth retardation that is not clearly noticeable until 2–3 months of cultivation. In order to study modes of HPM transmission and to test possible control measures, a laboratory challenge model was needed that would mimic the mode of infection in shrimp ponds.

**Results:**

We describe successful transmission in a cohabitation model with natural *E. hepatopenaei* (EHP)-infected shrimp in closed, perforated plastic containers placed in aquaria together with free-swimming, uninfected shrimp. After a period of 14 days all the free-swimming shrimp tested positive by PCR (approximately 60% with heavy infections evident by 1-step PCR positive test results) and gave positive histological and *in situ* hybridization results for *E. hepatopenaei* (EHP) in the hepatopancreas.

**Conclusions:**

A laboratory cohabitation model for studying *E. hepatopenaei* (EHP) has been developed and used to confirm that *E. hepatopenaei* (EHP) can be directly transmitted horizontally among shrimp via water. The model will facilitate studies on methods to prevent the *E. hepatopenaei* (EHP) transmission.

## Background

Two species of microsporidia have been reported so far as pathogenic to cultivated *Penaeus monodon* and *P. vannamei* in Thailand. These are *Agmasoma penaei* [1] and *Enterocytozoon hepatopenaei* (EHP) [2, 3]. In contrast to *E. hepatopenaei* (EHP) that does not cause any obvious gross signs of infection, *A. penaei* infections are characterized by a milky white appearance in the usually translucent musculature of the shrimp abdominal segments, sometimes referred to as “cotton shrimp disease” in English [4] or “white back disease” in Thai [1, 5, 6]. Microsporidian infections have been recorded in eight species of penaeid shrimp worldwide. [1, 7–16], but only two of them are currently known to infect cultivated shrimp in Thailand and its neighboring countries.

*A. penaei* cannot be transmitted horizontally among shrimp in rearing ponds by either spores or cannibalism [1, 5]. Since some fish species in Thailand gave PCR positive test results for *A. penaei* in Thailand [17, 18] a successful control strategy was devised that consisted of excluding fish (suspected to be intermediate hosts) from shrimp hatcheries and farms. Because of this practice, the prevalence of cotton shrimp disease (*A. penaei* infections) in cultivated shrimp in Thailand is very low.

*E. hepatopenaei* (EHP) was first described and characterized in 2009 from hepatopancreatic tissue of cultivated black tiger shrimp *P. monodon* in Thailand. However, it had first been reported as a previously uncharacterized microsporidian in juvenile shrimp in 2001 [19] during studies attempting to determine the cause of retarded growth in cultivated *P. monodon*. At that time, results for detection of *E. hepatopenaei* (EHP) by the presence of spores using light microscopy and confirmation by *in situ* hybridization did not correlate statistically with the occurrence of retarded growth *E. hepatopenaei* (EHP) morphologically resembles previously reported, unidentified microsporidians from hepatopancreatic tissue of *P. monodon* in Malaysia [20] and *P. japonicus* in Australia [21], suggesting that *E. hepatopenaei* (EHP) may be endemic in Australasia and have the capability of infecting multiple species of shrimp. Thus, after introduction and wide-spread cultivation of exotic whiteleg shrimp (*P. vannamei*) in Asia, outbreaks of HPM caused by *E. hepatopenaei* (EHP) in cultivation ponds were also reported [3, 22]. This was despite the fact that post-larvae used to stock some of the ponds were originated from specific pathogen free (SPF) broodstock free from *E. hepatopenaei* (EHP). In pond reared juvenile shrimp, *E. hepatopenaei* (EHP) infects only tubule epithelial cells of the central region of the hepatopancreas and does not extend into the embryonic cell region (E-cells) [2, 22]. It can sometimes also be seen in cells of the midgut epithelium near its junction with the hepatopancreas.

With respect to disease control in shrimp cultivation ponds, the most important difference between *E. hepatopenaei* (EHP) and *A. penaei* is the fact that *E. hepatopenaei* (EHP) can be horizontally transmitted among shrimp in rearing ponds by cannibalism [3] while *A. penaei* cannot [1]. This makes control in rearing ponds more difficult, and it raised the question as to whether transmission among shrimp in rearing ponds might occur also via *E. hepatopenaei* (EHP) spores released into the water in shrimp feces. We reasoned that if transmission via spores in water did occur, then cohabitation of *E. hepatopenaei* (EHP)-infected shrimp with uninfected shrimp would be successful and could be used as a challenge model for testing preventative measures against HPM in the laboratory. Here we describe a successful challenge model for transmission of *E. hepatopenaei* (EHP) in the laboratory by cohabitation of infected shrimp with uninfected shrimp.

## Methods

### Collection of infected shrimp and naïve shrimp

Naïve juvenile *P. vannamei* were acquired together with rearing water from a shrimp nucleus breeding center, while juvenile *P. vannamei* infected with *E. hepatopenaei* (EHP) were collected together with rearing water from cultivation ponds in and around Bangkok, Thailand. All were transported to the laboratory in plastic containers with proper aeration. They were acclimated at Centex Shrimp in 100 L tanks containing artificial seawater (Marinium Reef Sea Salt, Mariscience Intl. Co. Ltd, Buenos Aires). From each shrimp lot, 10 samples were arbitrarily selected to test by nested PCR [3] for the presence of *E. hepatopenaei* (EHP) in hepatopancreatic tissue (sufficient to detect its presence at 26% in the target population with 95% confidence) [23] to confirm infection in the case of infected shrimp and to confirm absence in the case of naïve shrimp. Since the infected shrimp and naïve shrimp were raised at different salinities (10 ppt and 25 ppt, respectively), salinity for all shrimp was adjusted during acclimatization in artificial seawater to 20 ppt by increasing or decreasing the salinity at 2 ppt/h.

### Cohabitation assays

**Fig. 1:**
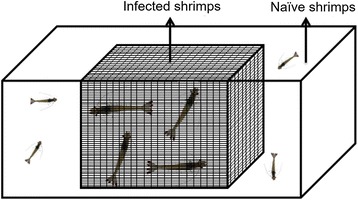
Schematic drawing (not to scale) of the setup for the cohabitation challenge experiment. In the diagram, naïve shrimp are outside the basket cage and infected shrimp inside. However, the process was also reversed with the same test outcome

All cohabitation tests were carried out at 20 ppt. Two aquaria containing 150 L artificial seawater each were set up with 10 infected (10–15 g) juvenile shrimp and 10 naïve post larvae 35 (PL_35_) (2–4 g) per aquarium. The naïve and infected shrimp were separated by having one or the other placed in closed plastic baskets to prevent contact between the two groups (Fig. 1). The shrimp were fed with commercial feed pellets twice a day until the end of the experiment. Three naïve shrimp from each tank were sampled at 7 days and 14 days post cohabitation. Each shrimp was dissected to reveal the hepatopancreas, a small portion of which was put in lysis buffer for DNA extraction. The entire remainder of the whole shrimp was fixed with Davidson’s fixative and processed for histopathological analysis by light microscopy following standard methods [24]. Two negative control groups were setup similar to the above mentioned groups, with 10 naïve shrimp inside the basket cages and 10 naïve shrimp outside.

### DNA extraction and PCR detection of *E. hepatopenaei* (EHP)

Total DNA was extracted according to the traditional phenol-chloroform method [25]. Hepatopancreatic tissue was homogenized prior to DNA extraction in lysis buffer (50 mM Tris–HCl, pH 9.0, 100 mM EDTA, pH 8.0, 50 mM NaCl, 2% SDS and 10 μg/ml proteinase K). *E. hepatopenaei* (EHP) was detected using a previously reported nested PCR protocol targeting its SSU rRNA gene [3] yielding amplicons of 779 and 176 bp (Table 1).

**Table 1:** Primer sequences used for PCR amplification and *E. hepatopenaei* (EHP) DIG probe preparation

Primer name	Sequence (5′-3′)	Amplicon (bp)	Reference
SSU-PCR 1^st^ PCR			Tangprasittipap et al. 2013
ENF779 ENR779	CAGCAGGCGCGAAAATTGTCCA A AGAGATATTGTATTGCGCTTGCT	779	
Nested PCR			
ENF176 ENR176	CAACGCGGGAAAACTTACCA ACCTGTTATTGCCTTCTCCCTCC	176	
EHP DIG probe			This paper
ENF411	AGGTGGTGTTAAAAGCCATTGAG	235	
ENR176	ACCTGTTATTGCCTTCTCCCTCC		

### Preparation of a DIG-labelled DNA probe for *E. hepatopenaei* (EHP)

A DNA probe for *in situ* detection of *E. hepatopenaei* in shrimp tissue sections was prepared by PCR using primers ENF411 and ENR176 targeting the SSU rRNA gene (Table 1) in a PCR reaction using a DIG-labelling mix (Roche, Germany) that contained digoxigenin-11-dUTP (DIG-11-dUTP) to substitute for a portion of the dTTP in the dNTP mix. The PCR reaction mix contained 1X PCR buffer, 1.5 mM MgCl_2_, 0.2 μM primers, 1X PCR DIG labelling mix and 1.25 unit Taq polymerase (Invitrogen). The PCR protocol consisted of initial denaturation at 95 °C for 5 min followed by 35 cycles of denaturation at 95 °C for 30s, annealing at 58 °C for 30s and extension at 72 °C for 45 s. After a final extension at 72 °C for 7 min, the reaction was cooled down to 16 °C. The DIG-labelled probe was then run on a 1.2% agarose gel and the probe band was cut for gel extraction using a NucleoSpin Gel and PCR clean-up kit (Macherey-Nagel, Germany) according to the manufacturer’s instructions. The concentration of the probe was measured using a Qubit fluorometer (Invitrogen, USA).

### *In situ* nucleic acid hybridization using the DIG-labelled probe

Paraffin embedded tissue sections were de-paraffinized in xylene and rehydrated before being treated with 5 μg/ml proteinase K (Invitrogen, USA) in 1X TNE buffer for 10 min at 37 °C. The sections were then incubated with 150 μl pre-hybridization buffer [4X SSC containing 50% (v/v) deionized formamide] at 37 °C for 30 min after which the sections were covered with the DIG-labelled probe (200 ng/slide) in hybridization buffer [containing 50% deionized formamide, 5% dextran sulfate, 1X Denhardt’s solution (Sigma), 4X SSC, 250 μg/ml salmon sperm DNA (Invitrogen)]. A cover glass was added followed by incubation at 42 °C for at least 16 h. This was followed by high stringency washes and equilibration in buffer I (100 mM Tris–HCl and 150 mM NaCl, pH 7.5), followed by incubation in buffer II [Buffer I containing 0.5% Blocking reagent (Roche, Germany)] for 30 min at RT. After the incubation, a solution containing alkaline phosphatase conjugated anti-digoxigenin (1:500) was added for 1 h. The unbound antibody was washed off twice using 1X buffer I before the sections were equilibrated in buffer III (100 mM Tris–HCl, 100 mM NaCl and 50 mM MgCl_2_, pH 9.5). Signal was developed by addition of NBT-BCIP substrate (Roche, Germany) and colour development was followed by microscopy before stopping, counterstaining with Bismarck brown Y (Sigma, USA) and dehydrating for preparation of permanent slides.

## Results

### Preliminary PCR screening

All samples of 10 shrimp from all batches of naïve shrimp tested gave negative results by nested PCR analysis for *E. hepatopenaei* (EHP). At the same time, all of the samples of purported *E. hepatopenaei* (EHP)-infected shrimp gave positive results by the same test, showing either a single nested PCR amplicon of 176 bp (relatively light infections) or two amplicons (severe infections), one of 779 bp for the first-step PCR and one of 176 bp for the second, nested PCR step.

### Cohabitation bioassay

**Fig. 2:**
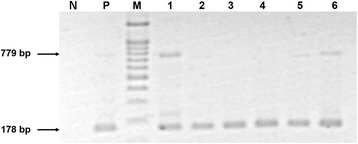
Agarose gel showing PCR amplicons for the SSU rRNA gene of *E. hepatopenaei* (EHP) in naïve shrimp at 14 days after cohabitation with *E. hepatopenaei* (EHP)-infected shrimp. Note that 4 of the samples gave amplicons for the first-step of the nested PCR assay, indicating severe infections. The gel image has been inverted to make first step PCR band in Lane 2 and 5 more prominent. N: Negative control, P: Positive control, M: Marker, Lanes 1–6: shrimp samples

During the cohabitation experiments, no mortality was expected and none occurred, since HPM is not usually associated with mortality. For the 6 control shrimp sampled (3 each from each of two aquaria) none gave positive nested PCR assay results for *E. hepatopenaei* (EHP). In addition, none of the 6 specimens from day 14 gave positive *in situ* hybridization results for *E. hepatopenaei* (EHP). By contrast, all 6 shrimp samples taken from the *E. hepatopenaei* (EHP) cohabitation aquaria at day 14 were positive for *E. hepatopenaei* (EHP) by the nested PCR assay, 4 at the first PCR step (indicating severe infections) and 2 at the nested step only (indicating light infections) (Fig. 2).

### Histopathology by H & E staining and *in situ* hybridization assays

**Fig. 3:**
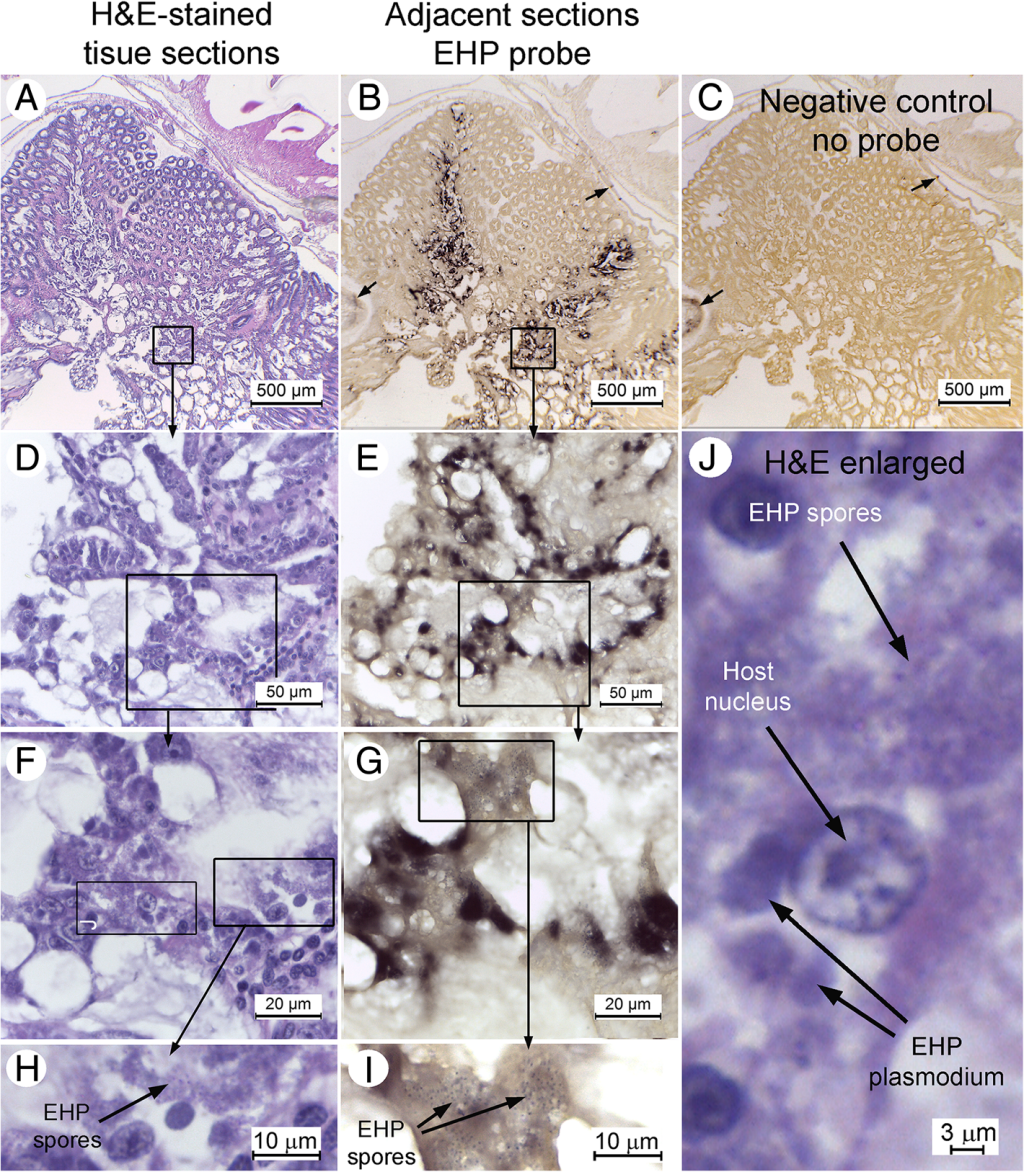
Progressively magnified photomicrographs of adjacent HP tissue sections stained with H&E (Column 1) or showing *in situ* hybridization results using a DIG-labeled probe for *E. hepatopenaei* (EHP) (Column 2). **a** Low magnification (10x objective) showing that *E. hepatopenaei* (EHP) cannot be resolved by H&E staining. **b** Adjacent tissue section showing that focal areas of *E. hepatopenaei* (EHP) infection can be easily detected by a DIG-labeled probe. **c** No probe negative control showing some regions of non-specific signal (*arrows*) also present in (**b**) (similar arrows). **d** Higher magnification (40x objective) of the area outlined in (**a**) showing that *E. hepatopenaei* (EHP) still cannot be easily resolved. **e**Adjacent section showing that *E. hepatopenaei* (EHP) spores cannot be easily resolved by the DIG probe. **f** Higher magnification (100x objective) of the area outlined in (**d**) showing spores and intracellular plasmodia of *E. hepatopenaei* (EHP) that are just visible. Squares outline regions that are magnified in (**h**) and (**j**) to make the spores and plasmodia easier to see. **g** and **i** Adjacent sections showing a magnified region of (**g**) making it easier to see *E. hepatopenaei* (EHP) spores labeled by the DIG probe

H&E stained sections of HP tissue from the PCR positive cohabitated shrimp specimens showed varying levels of infectivity. Some samples showed extensive histological signs corresponding to *E. hepatopenaei* (EHP) developmental stages such as plasmodia and spores (Fig. 3), albeit under oil immersion, whereas some samples gave no histological signs of infection, contradicting the PCR results in section 3.2. However, the DNA probe specific for the SSU rRNA gene of *E. hepatopenaei* (EHP) gave positive hybridization results revealing that many of the HP tubule epithelial cells were infected (Fig. 3). This confirmed the *E. hepatopenaei* (EHP) infections indicated by the PCR results, even though the samples showed no clear developmental stages such as plasmodia and spores by H&E staining in the single tissue sections examined for each specimen. Although tedious and time consuming, the *in situ* hybridization method provided a more reliable and accurate assessment of the severity of infection than did H&E staining.

## Discussion

The successful cohabitation model described herein overcomes the disadvantage of the previous destructive, oral challenge model using *E. hepatopenaei* (EHP)-infected hepatopancreatic tissue [3]. It allows for the testing of shrimp broodstock and other potential carriers for infection with *E. hepatopenaei* (EHP) in a non-destructive manner. It also allows for the testing of water or feed treatments for possible efficacy in protecting against *E. hepatopenaei* (EHP) infection via this natural infection route. Protection might result from treatments to kill spores or to interfere with their process of polar tube extrusion that must occur for the spore contents to enter host cells [26]. The cohabitation model will also be useful for testing potential carriers of *E. hepatopenaei* (EHP) in both directions, i.e., from shrimp to potential carriers and vice versa. At the same time, the previous method of oral challenge using infected HP tissue [3] will still be useful for testing such things as *E. hepatopenaei* (EHP) inactivation by heating, chilling, freezing, etc.

The main reason we developed this cohabitation method was that spores we had purified by density gradient centrifugation from hepatopancreatic tissue of infected juvenile shrimp following previously published methods [2] failed to induce infections when used by bath exposure, when added to shrimp feed or when administered by reverse gavage (unpublished). The reason for these failures is still unknown. It is possible that the steps in purification by density gradient separation or that refrigeration of the purified spores at 4 °C inactivated them prior to use in those tests. To address these issues, we attempted to test for viability of the purified, refrigerated spores using previously published methods for vital staining [27] but these failed for reasons including stain uptake by the spore walls, or auto-fluorescence of the spore walls (unpublished). We also tried to induce polar tube extrusion by previously published methods [28], but these tests also failed, possibly indicating that the refrigerated spores were indeed non-viable.

Another possibility is that the spores produced at different host life stages may differ in ability to re-infect the same host. This phenomenon has been previously reported for other microsporidians [29] where spores produced at a later host developmental stage may differ physiologically from those produced at an earlier host stage and that such spores may require some kind of activation step such as drying before they become infectious. It is also possible that spores produced at a later host developmental stage may be destined for an alternative host species. These two possibilities seem unlikely for *E. hepatopenaei* (EHP) since our cohabitation tests employed infected juvenile shrimp similar in age to those used for spore purification. On the other hand, it is also possible that the juvenile shrimp may produce two different types of spores, one for horizontal transmission and another for transfer to an alternative host. Clearly, much more work is needed on this emerging pathogen to elucidate its host range and life stages.

In addition, during the development of the cohabitation model, our team noticed that assessment of EHP infection purely by the presence of spores by light microscopy can be misleading. Work using an *in situ* hybridization probe specific for *E. hepatopenaei* (EHP) with cultivated *P. vannamei* sometimes reveal severe infections (approximately 75% hepatopancreatic tissue involvement) with few recognizable intracellular stages of plasmodial development and spore production, as assessed by histological analysis of tissue sections [22, 30]. Hence, as demonstrated in this study, *in situ* hybridization assays with the same tissue section were performed in parallel with the standard H&E analysis to better estimate the extent of infection. Nonetheless, the histopathological assay is a tedious and time-consuming process. We, therefore, recommend instead that PCR based on DNA extracts from homogenates of hepatopancreatic tissue of individual or pooled shrimp samples be used for initial *E. hepatopenaei* (EHP) diagnosis [3, 16, 31]. Moreover, based on current results indicating that the degree of growth retardation from *E. hepatopenaei* (EHP) is positively related to pathogen load, it is probably best to test for *E. hepatopenaei* (EHP) using quantitative PCR methods ([32], our unpublished work).

## Conclusions

The bioassay for inducing laboratory infection is a primary requirement for studying any newly emerging pathogen and our co-habitation model is straightforward and simple. It can be applied to develop protective treatments for water or feed and to test *E. hepatopenaei* (EHP) transmission between shrimp and suspected carriers. In the meantime, the problems associated with the use of purified spores may be solved allowing for additional bioassays related to issues such as infectious spore dose.

## Abbreviations

EHP: *Enterocytozoon hepatopenaei*
NBT-BCIP: HP, Hepatopancreas
